# Late Fontan Circulatory Failure. What Drives Systemic Venous Congestion and Low Cardiac Output in Adult Fontan Patients?

**DOI:** 10.3389/fcvm.2022.825472

**Published:** 2022-03-14

**Authors:** Alexander Van De Bruaene, Guido Claessen, Thomas Salaets, Marc Gewillig

**Affiliations:** ^1^Division of Cardiology, Department of Cardiovascular Sciences, University Hospitals Leuven, KU Leuven, Leuven, Belgium; ^2^Division of Pediatric Cardiology, Department of Cardiovascular Sciences, University Hospitals Leuven, KU Leuven, Leuven, Belgium

**Keywords:** univentricular heart, Fontan circulation, cavopulmonary connection, Fontan circulatory failure, heart failure

## Abstract

The Fontan circulation provides definite palliation for children born with a single anatomical or functional ventricle by diverting systemic venous blood directly to the pulmonary arteries, effectively rendering systemic venous return into portal vessels to the lung. Although this restores pulmonary blood flow and avoids the mixture of oxygenated and deoxygenated blood, it also results in elevated systemic venous pressures and low cardiac output. These are the two hallmarks of any Fontan circulation and the cause of Fontan circulatory failure later in life. We highlight the determinants of systemic venous return, its changed relationship with the pulmonary circulation, how it affects preload, and the changed role of the heart (myocardium, valves, and heart rate). By critically evaluating the components of the Fontan circulation, we hope to give some clues in how to optimize the Fontan circulation and avenues for future research.

## Introduction

Francois Fontan and Eugene Baudet's pioneering work on the surgical treatment of patients with tricuspid atresia eventually led to the development of the Fontan operation (1). The surgeons' main aim was to restore pulmonary blood flow and to eliminate the mixture of venous and oxygenated blood. Whilst this remains crucial to the concept of the Fontan circulation, Drs Fontan and Baudet's hypothesis that this required some form of an atrial (or ventricular) “pump” was later superseded when de Leval nicely illustrated that the right atrium as a valveless chamber does not contribute to blood flow at the higher venous pressures observed in Fontan patients. The right atrium also has no reservoir function, rather causing energy loss than contributing to hemodynamic efficiency (2). The use of computational fluid dynamics avant-la-lettre to demonstrate the advantage of the total cavopulmonary connection over the atriopulmonary connection is testimony to the innovation that has made advancements in the field of congenital heart disease possible.

While the Fontan operation became the final palliation for thousands of patients with a single anatomical or functional ventricle worldwide, pediatric and adult congenital cardiologists are increasingly confronted with the limitations posed by the circulation created many years ago (3–5). There is no doubt that the surgical intervention has dramatically improved the longevity and quality of our patients' lives, but long-term morbidity and mortality remain high (4). Exercise intolerance, Fontan-associated liver disease, protein loosing enteropathy, plastic bronchitis, arrhythmia, thrombo-embolic complications, and neuro-cognitive limitations are just a few of the complications related to Fontan circulatory failure that clinicians are increasingly seeing in clinical practice.

The extent of the definition of “Fontan circulatory failure” recently proposed in ESC Heart Failure also underscores our lack of knowledge with regards to Fontan physiology and likely also translates into a lack of effective treatment options for Fontan circulatory failure (6). As Fontan, Baudet, Marcelletti, and de Leval did in the past, there is an urgent need for innovative solutions improving palliation of single ventricle physiology and tackling the complications unintentionally caused by the construction of a Fontan circulation. Although we have published a more theoretical approach to conceptualize the Fontan circulation (5), the aim of this introductory article is to present our current understanding of Fontan physiology and the different sources and components of Fontan attrition over time into adult life. There is no doubt that every Fontan circuit constructed bears in itself the components of its own failure. But since innovation follows understanding based on simple observation, the case of the failing Fontan is not necessarily hopeless.

## Creating the Fontan Circulation

De Leval's observations paved the way for the modern version of a Fontan circuit: the total cavopulmonary connection (2). In the neonatal period and early infancy, interventions guarantee adequate systemic flow (coarctectomy, Damus-Kaye-Stansel, or Norwood arch repair in case of obstruction) while simultaneously balancing pulmonary blood flow (banding or shunt). This is followed by diversion of systemic venous return from the superior caval vein to the pulmonary artery at the age of 3–9 months (bidirectional Glenn or partial cavopulmonary shunt), followed by Fontan completion at the age of 2–4 years (connection of the inferior caval vein to the pulmonary artery) (7).

From the beginning, it was clear that not all Fontan circulations would be created equal. For example, a patient starting off with slightly higher pulmonary vascular resistance, pulmonary artery hypoplasia, distortion of the pulmonary arteries, AV valve regurgitation, or ventricular dysfunction will likely have worse Fontan physiology after palliation. Maybe more significantly, this also stresses the importance of a carefully and considerate construction of the Fontan circulation and especially its most limiting building block: pulmonary circulation. Allowing sufficient growth of the pulmonary arteries prior to referring for a Glenn may be crucial if one considers the implications of small changes in pulmonary vascular resistance on cardiac output and systemic venous pressure in a Fontan patient as outlined below (8–10).

What is in essence an extra-cardiac operation nevertheless has dramatic consequences for the cardiovascular system. The Fontan circuit consists of the surgical venous connection and the graft, the pulmonary arteries, the pulmonary capillaries, and pulmonary veins and renders the systemic venous return into portal vessels to the lung (5). Since systemic venous return is connected directly to the pulmonary arteries without a subpulmonary ventricle, the Fontan circuit imposes an additional flow restriction, causing upstream congestion and decreased downstream flow (11). The main function of the Fontan circuit is to generate sufficient hydraulic power to overcome the resistance of the pulmonary circulation at rest and during exercise.

## Physiology of the Fontan Circulation

### Venous Return: In the Driver's Seat

#### Fontan at Rest

It remains remarkable that venous pressure in the Fontan portal system is sufficient to generate pulmonary blood flow. In ideal circumstances, a mean pulmonary arterial pressure of (at least) 15 mmHg is required to keep the pulmonary vasculature patent (i.e., higher than alveolar pressure and distal pulmonary venous pressure) (12). In a Fontan patient, this is accomplished by a combination of mainly passive and weakly active forces [peripheral muscle contraction (13), respiratory inspiration (14, 15), and downward displacement of the atrioventricular valve expanding atrial volumes (12)]. We must also consider that the absence of a subpulmonary ventricle in the Fontan circulation has additional consequences such as a lack of dilatation and recruitment of pulmonary blood vessels, lack of kinetic energy, asymmetric pulmonary perfusion, and loss of pulsatility. Loss of pulsatility may further increase the energy necessary to propel blood through the pulmonary vasculature. Since normally one third of the energy generated by the right ventricle is absorbed by the pulmonary blood vessels in systole and restituted in diastole to maintain patency of the distal vessels, it follows that pulmonary impedance increases when hydraulic power converts into a pure pressure gradient as is the case in the Fontan circulation.

Second, since the superior caval vein (±30% of venous return), inferior caval vein (±45% of venous return), and hepatic veins (±25% of venous return) now function as portal vessels to the lungs, factors determining systemic venous return will determine filling of the systemic ventricle and eventually cardiac output. Macé et al. described that such a circulation results in a downward shift of the venous return curve (16). As this would result in decreased venous return, not matching (required) cardiac output, blood volume increases as a physiologic adaptation to the Fontan state. This increases cardiac output at the expense of an increase in systemic venous pressures and increased overall blood volume. Simultaneously (and advantageously), the higher systemic venous pressure will result in recruitment of pulmonary blood vessels, hence lowering pulmonary vascular resistance.

**Figure 1:**
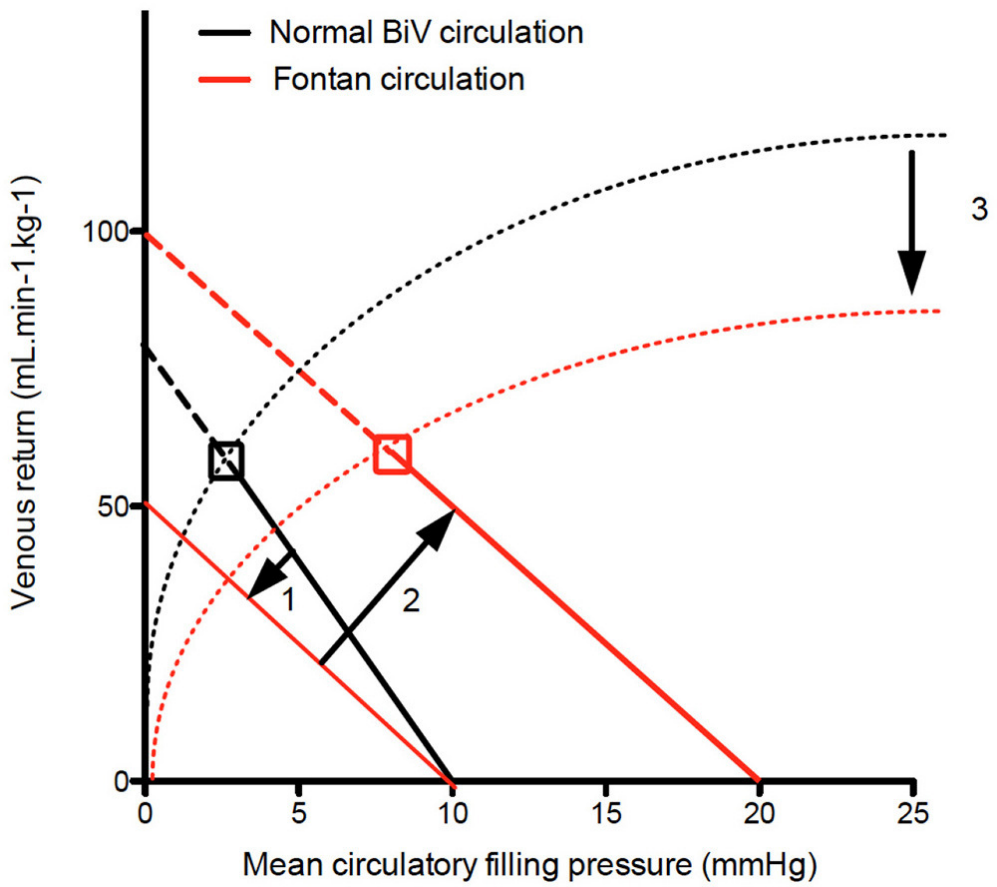
Venous return curve. In Fontan patients the venous return curve shows on downward rotation due the pulmonary vasculature (and hence PVR) being incorporated in venous return (1). Volume load brings back venous return at the level of biventricular circulation at the expense of an increase in mean circulatory filling pressure (2). Finally since left atrial pressure is the backpressure for venous return, increased left atrial pressure will result in decrease cardiac output. The dashed lines indicate the Frank Starling curve, indicating lower contractility (3).

Third, there is a shift of the mean circulatory filling pressure, which is usually located at the level of peripheral small veins and venules to the pulmonary vasculature. With the atrial pressure as back pressure, the venous resistance is now similar to pulmonary vascular resistance. This shift illustrates the importance of the pulmonary circulation and how it functions as a dam causing congestion upstream and low flow downstream, the two hallmarks of Fontan physiology (16). From this it comes to reason that factors affecting mean filling pressure (blood volume and venous muscle tone), atrial pressure (atrial compliance, contractility, and valve competence and resistance but also ventricular filling pressures) and venous resistance (autonomic tone, muscle pump, intra-abdominal pressure, flow inefficiency, pulmonary artery dysplasia, and pulmonary vascular resistance) will affect venous return (Figure 1).

#### Fontan During Exercise

**Figure 2:**
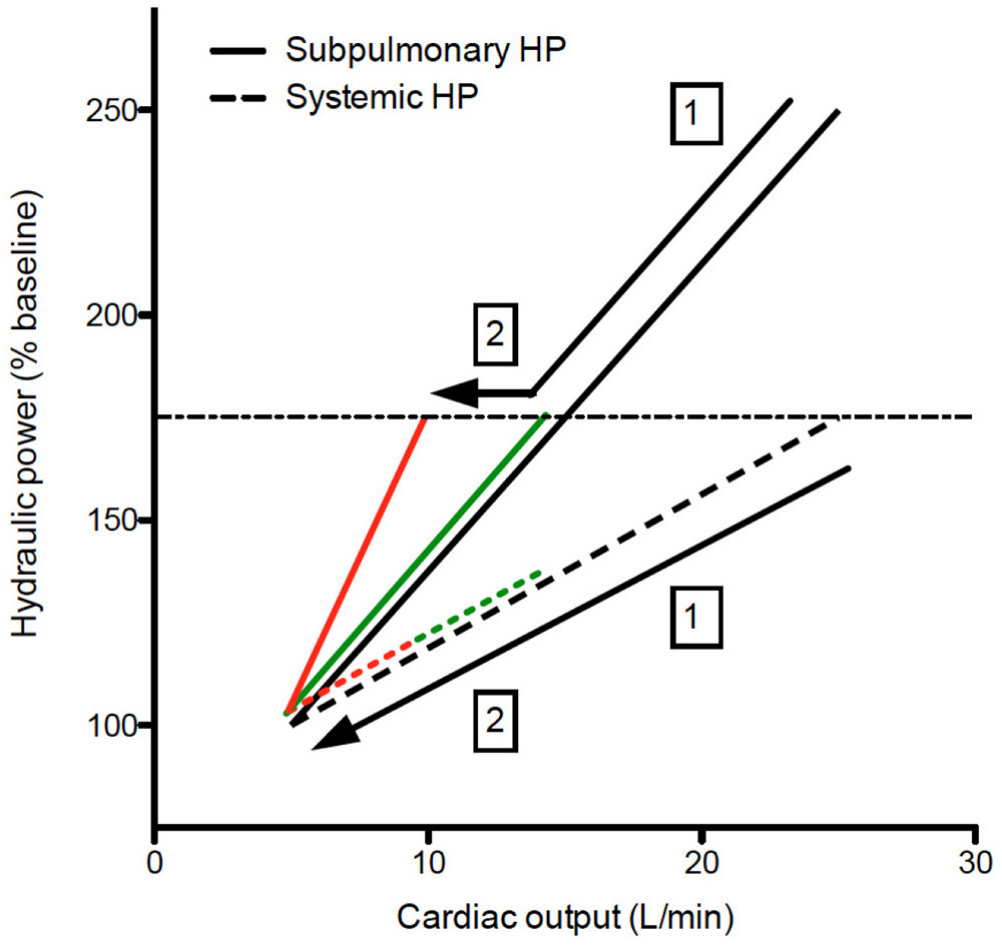
Hydraulic power relative to cardiac output during exercise. Both on healthy controls and Fontan patients, the increase in hydraulic power as expressed relative baseline to augment cardiac output is larger on the subpulmonary ventricle, even in NYHA Fontan patients (green lines), the increase in hydraulic power (and hence cardiac output) is limited (1). NYHA ≥ 2 Fontan patients (red lines), a steeper increase in hydraulic load will further limit the increase in cardiac output during exercise (2). The dashed horizontal line represent hemodynamic reserve of the Fontan patients, which is limited due to the absence of a subpulmonary ventricle. Black line, controls; Green lines, NYHA 1 Fontan; Red lines, NYHA ≥ 2 Fontan.

Exercise capacity is determined by the ability of the cardiorespiratory system to deliver oxygenated blood to the working muscles, thereby providing substrate for energy generation. Normally the main factor determining oxygen delivery is the cardiac output (CO) generated by the hydraulic power of each ventricle (17). In a normal circulation right atrial pressure (and systemic venous pressure) changes little during exercise (18), making systemic venous return largely independent of “afterload” because of the intervening right ventricle. In a Fontan circulation, there is no subpulmonary pump, so systemic venous return (provided by the peripheral muscle pump, venous compliance, and venomotor tone) is responsible for providing the work necessary to augment flow. Prior research has consistently demonstrated that very little work is required to generate CO against the vascular load of the pulmonary circulation under resting conditions (see also paragraph above) (19, 20). This is also the condition sine qua non for a working Fontan circulation. However, during exercise, pulmonary artery pressure normally increases in a near-linear manner such that there is a dramatic increase in the work required by the subpulmonary pump in order for CO to augment normally (21–23). Moreover, intensive exercise training results in disproportionate subpulmonary cardiac remodeling, highlighting the disproportionate effect of exercise on the subpulmonary pump (24). Insufficient systemic venous return augmentation during exercise in Fontan patients due to limitations in the ability to increase systemic venous pressures against pulmonary vascular load is the primary source of exercise limitation in a Fontan circulation. Indeed, Egbe et al. clearly indicated that steeper pressure-flow plots (independent of the cause) are associated with worse exercise tolerance (25). But their study in poor Fontan patients and our earlier study in “good” Fontan patients also highlights the significant limitations in CO augmentation (i.e., CO at peak exercise) during exercise when compared to a normal biventricular system (25, 26) (Figure 2).

It is well-known that ventricular and stroke volumes oscillate with respiration. In a normal biventricular physiology this results in peak right and left ventricular volumes (and stroke volume) at peak inspiration and expiration, respectively, a differential effect which is maintained throughout exercise (27). In a Fontan circulation, Inferior caval vein flow shows marked respiratory variability with inspiratory facilitation and expiratory inhibition, which becomes less evident during exercise (28). So aside from maintaining a low systemic venous pressure (at rest and during exercise), providing sufficient hydraulic power to increase CO (during exercise), the right heart and pulmonary circulation are important to buffer venous return and keep left ventricular stroke volume constant (at rest but especially during exercise). Indeed, we have shown that the respiratory pump in Fontan patients causes a respiratory-induced variation in stroke volume (which appears to exacerbated during exercise) which is only partly attenuated by the pulmonary circulation (15).

The paragraphs above delineate the importance of systemic venous return for a well-functioning Fontan circulation. They also suggest considering the place of the pulmonary circulation which renders superior and inferior caval vein as well as hepatic veins into portal vessels toward the pulmonary circulation with its advantages (pulmonary blood flow) and disadvantages (increased systemic venous pressure, decreased cardiac output, and increased stroke volume variation at rest but especially during exercise). In doing so, preload to the ventricle which is usually abundant, becomes limited and systemic venous return through the pulmonary circulation becomes the main determinant in the regulation of pulmonary blood flow and hence exercise capacity.

Understanding the importance of the Fontan portal circuit for a well-functioning Fontan circulation may also give us some clues in how to optimize the Fontan circulation.

**Figure 3:**
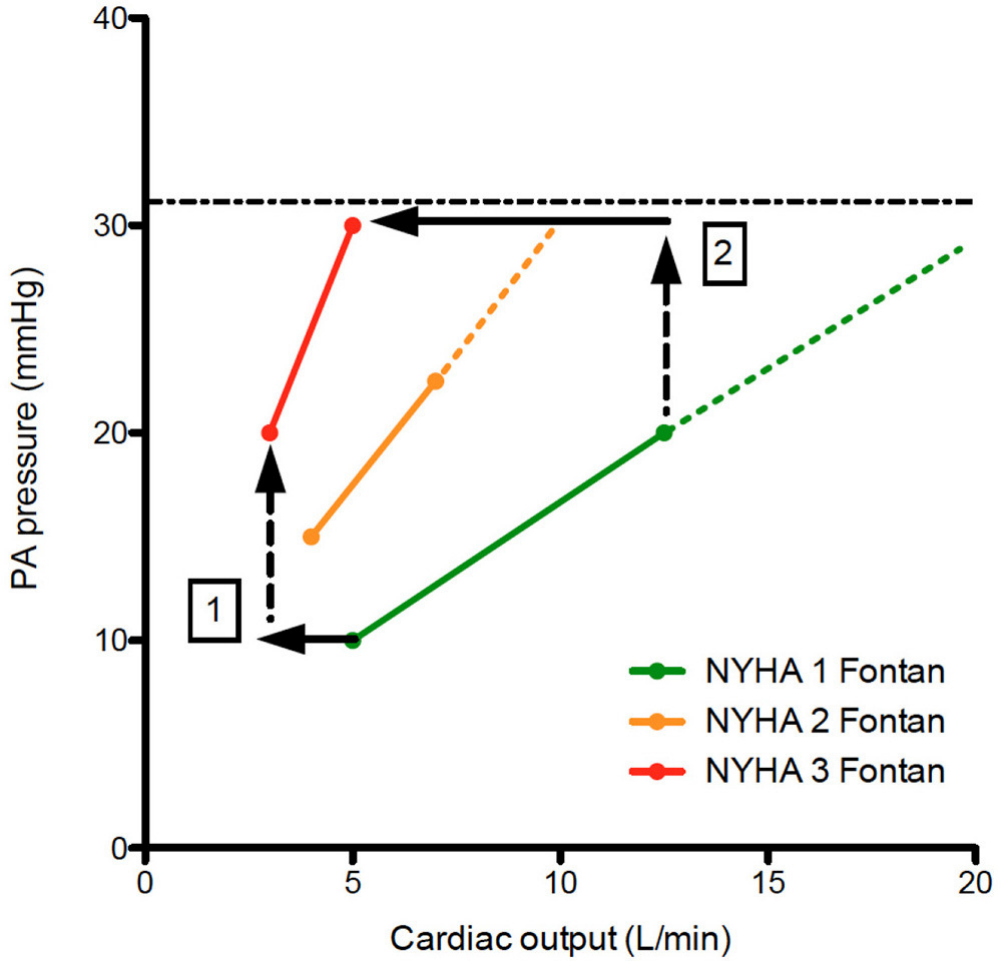
Pressure flow plots indicating the increase in PA pressure vs. cardiac output during exercise. When comparing NYHA 1 Fontan patients with NYHA 2&3 Fontan patients, note there is a decrease in cardiac output despite increase in resting PA pressure (1). During exercise, the increase in PA pressure is steeper for NYHA 2&3 patients. A hypothetical maximum of PA pressure (systematic venous pressure) results is an exacerbated decrease in cardiac output resulting in significant functional limitation of the Fontan patient (2). The dashed lines represent hemodynamic reserve of the Fontan patient.

Power loss in the TCPC has been a point of intensive research since pressure gradients across surgical connections, uneven distribution of pulmonary flow to the lungs, and collision of flow is disadvantageous for the Fontan circulation (29, 30). Offsetting of the caval anastomoses was recognized early on, and adaptations for the Fontan connection reducing power loss have been suggested. Oftentimes, the size of the Fontan conduit (either small from the beginning, or reduced in size whilst aging) may result in suboptimal hemodynamics which will be exacerbated during exercise as assessed by 4D MRI flow (31–33). Likely optimizing the Fontan conduit early in life (allowing sufficient pulmonary artery growth and additional shunt if needed) and later in life (stenting of the conduit to adult size or stenting of a hypoplastic left pulmonary artery if present) are required to maintain optimal Fontan hemodynamics as long as possible (5). Splanchnic vasoconstriction and decreased venous compliance are present in a well-functioning Fontan circulation, but failure of these compensatory mechanisms has been observed (34, 35). Regular exercise (36), compression stocking in case of varicose veins, and paracentesis in case of tense ascites may be of use in selected patients. Pulmonary vasodilators have been studied in patients with a Fontan circulation but the overall net clinical benefit in the two largest randomized controlled trials (FUEL trial and TEMPO trial) with an improvement in peak oxygen consumption of 3–5% has been limited so far (37, 38). Most centers (including ours) would advocate diuresis in case of Fontan circulatory failure with elevated systemic venous pressures, but the effects of decreasing overall blood volume in a preload dependent circulation requires more study (especially on its early and long term effect on cardiac output) (3). Furthermore, the extent to how much systemic venous pressure can increase during exercise requires further study, since this may pose a natural limitation to exercise capacity in Fontan patients (18, 26) and explain exacerbated reductions in peak CO in failing Fontan patients (Figure 3).

### The Heart: The Co-pilot as Bystander

#### A Preload Deprived Heart

Most cardiac lesions and most cardiac patients are characterized by cardiac dysfunction due to pressure and/or volume overload of the systemic or subpulmonary ventricle. But there is remarkably little evidence evaluating the effect of chronic volume deprivation on the ventricle (5). The paragraphs above illustrate why the systemic ventricle in a Fontan circulation lacks preload at rest and during exercise. Since there is no subpulmonary ventricle, even low-level changes in pulmonary vascular resistance (PVR) cause significant changes in cardiac output. Indeed, Egbe et al. showed that Fontan patients who have low cardiac index, measured during cardiac catheterization or echocardiography, coupled with higher PVR had the highest risk for Fontan circulatory failure (39, 40).

#### The Myocardium

**Figure 4:**
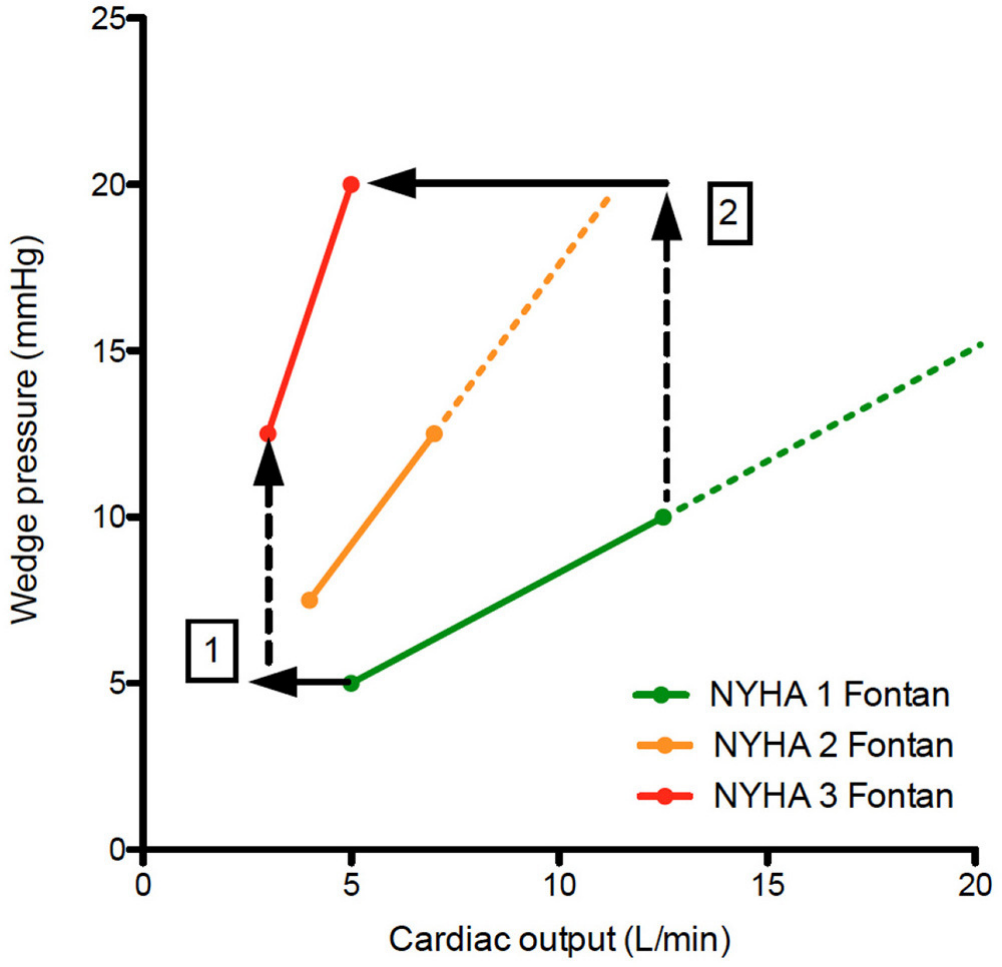
Pressure flow plots indicating the increase in wedge pressure vs. cardiac output during exercise. When comparing NYHA 1 Fontan patients with NYHA 2&3 Fontan patients, note there is a decrease in cardiac output despite increase in wedge pressure (1), which becomes more pronounced during exercise (2). This is counterintuitive in a preload dependent circulation. Diastolic dysfunction (which may be a consequence of preload deprivation) will exacerbate systemic venous hypertension and cardiac output limitation during exercise.

The staged Fontan palliation is associated with significant changes in volume load to the ventricle which evolves from being volume overloaded (prior to the Glenn) to preload deprived (after Glenn and Fontan completion) (41). This and other factors (surgical insult) may result in ventricular dilation, eccentric hypertrophy [increased mass:volume ratio due to a decrease in ventricular volume with increased wall thickness (42)] and could cause systolic and/or diastolic dysfunction (43). Indeed, systolic and diastolic dysfunction has been observed prior and after the Fontan operation (44) and relate to the degree of volume load prior to Fontan completion (45) and even to outcome (46). Other studies did show a normal contractile response to dobutamine, suggesting that in most Fontan patients ventricular function is not the main factor limiting CO at rest or during exercise (47). However, it is also true that due to expanding indications for Fontan repair, borderline ventricles have been incorporated into Fontan circuits in more recent years. This, in combination with aging of the ventricle in older Fontan patients, will render ventricular dysfunction (systolic and diastolic) a more important issue in the years to come (26). Aging may even be accelerated in Fontan patients similarly to what has been observed in sedentary people where the left ventricle becomes stiffer (due to lack of exercise induced ventricular stretching) and evolves toward heart failure with preserved ejection fraction phenotype (48, 49). Increased afterload (which could contribute to systolic and diastolic deterioration) has been reported, but may be secondary to decreased output in order to maintain blood pressure (50). Moreover, studies evaluating Ace inhibitors in patients with a Fontan circulation have been negative so far (51, 52). Indications for Ace inhibitors in 2-ventricle circulations, such as the presence neurohormonal activation, greater than mild ventricular dysfunction or atrioventricular valvular regurgitation, or increased afterload, have not been replicated in the Fontan cohort and warrant a considered approach in prescribing these drugs for Fontan patients (ref Wilson). When evaluating data from the article by Egbe et al. on pressure-flow plots during exercise, Fontan patients with a steeper pressure-flow curve also have a steeper increase in pulmonary wedge pressure during exercise (25, 26). This is all the more surprising in a preload limited circulation where one would expect a decrease in wedge pressure during exercise (if these were normal ventricles) (26, 53). Moreover, in the absence of a subpulmonary ventricle, even low-level changes in left atrial pressures will cause significant changes in cardiac output as is evident in differences in peak CO between Fontan subgroups (Figure 4).

When creating a Fontan circulation, the pediatric cardiologist and congenital cardiac surgeon have made tremendous advances, especially when balancing adequate pulmonary artery growth whilst preventing excessive volume load to the ventricle (5, 8, 10). The importance of physical activity (which introduces pulsatility to the pulmonary vasculature and improves filling of the ventricle with stiffness-reducing stretching) as well as preventing the accumulation of risk factors for a heart failure with preserved ejection fraction phenotype (obesity, hypertension, diabetes) cannot be understated during the life trajectory of any Fontan patients. Improved assessment and understanding of diastolic function in patients with a Fontan circulation is an unmet clinical need that hampers innovation and should be addressed. Continuous (milrinone) or intermittent (levosimendan) infusion of inotropes can improve organ perfusion and reduce venous congestion. In a large series of Fontan patients undergoing transplantation, 74% of patients received inotropic support. The potential benefit of a combination of diuretics with inotropes (resulting in systemic and pulmonary vasodilation in combination with inotropy) to maintain a euvolemic state requires further study.

#### Heart Rate

**Figure 5:**
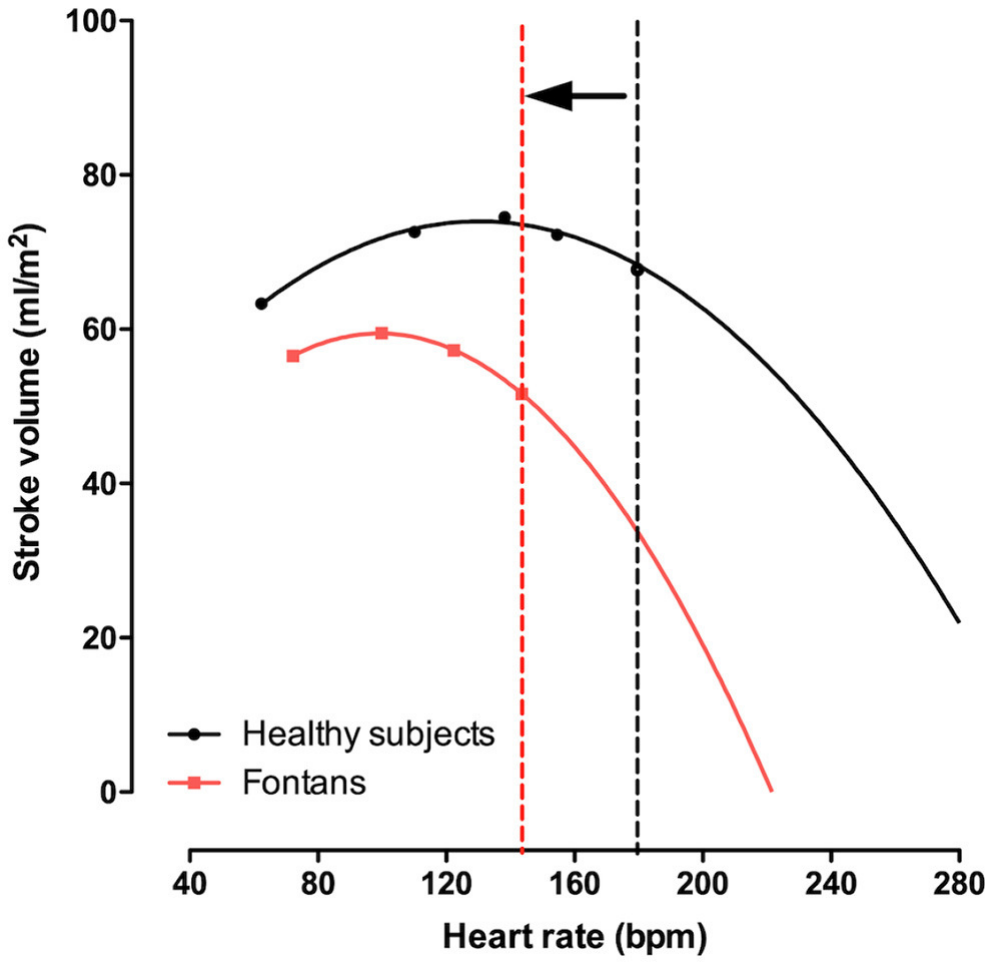
Stroke volume index vs. heart rate during exercise. In Fontan patient heart rate and exercise capacity is limited. In a significant proportion of Fontan patients, heart rate relative to workload is preserved (or even increased). Nevertheless, heart rate reserve is still reduced, which may be a physiologic mechanism preventing a full in stroke volume (and cardiac output). This highlighted in the figure, where the infliction point (vertical dashed line) beyond which a further increase in heart rate would result in falling cardiac output.

In Fontan patients the sinus node may be dysfunctional, either congenitally or damaged during multiple surgeries. Although chronotropic limitation has been extensively described in Fontan patients, prior studies have demonstrated that atrial pacing at rest does not augment CO, that at comparable exercise levels Fontan patients already have a faster heart rate, and that pacing beyond maximal heart rates does not improve exercise capacity (54–56). Whereas, CO augmentation in healthy controls is achieved by an increase in heart rate and stroke volume, in Fontan patients increase in CO is primarily achieved by increases of transpulmonary flow (or venous return). Indeed, increase in heart rate relative to metabolic demand is robust or even enhanced in Fontan patients. However, when stroke volume starts falling and CO plateaus, heart rate ceases to increase. All this suggests chronotropic constraint to prevent collapse should heart rate further increase (with falling stroke volumes) (57). The Bainbridge reflex could explain causality, but a direct feedback mechanism as CO cannot be maintained is another possibility (Figure 5).

A more aggressive rhythm control management strategy (including atrial pacing for junctional rhythms, DC cardioversion, ablation, and medical therapy) is warranted in Fontan patients. Not only would they not tolerate faster heart rates (58), preservation of atrial suction and contraction and optimizing diastolic filling time is important in preload dependent circulations (59).

#### The Atrioventricular Valve(s)

The atrioventricular valve(s) are often structurally abnormal in patients with a Fontan circulation, but functional atrioventricular valve regurgitation due to ventricular and/or annular dilatation, prior volume, and/or pressure overload has been described as well (60). Atrioventricular valve regurgitation is common, with about one fifth of patients presenting with moderate or severe atrioventricular valve regurgitation. Although its consequences have been well-described with a 2- to 3-fold increased risk of Fontan circulatory failure (61), its management after Fontan completion remains cumbersome (62). The highest risks for significant atrioventricular valve regurgitation are observed in patients with mitral atresia and a common atrioventricular valve and less frequently in patients with two valves or patients with tricuspid atresia (61). Experience from the Mayo clinic indicates that early intervention (before Fontan completion) has better outcome and that valve interventions after completion are associated with an increased risk of mortality or need for transplantation (63). Pathophysiology is straightforward, with increases in atrial pressure resulting in increased systemic venous pressures, lower cardiac output, and decreased reserve during exercise.

#### Neurohormonal State

Although we as clinicians feel that the interventions and altered Fontan circulatory hemodynamics trump all else, there is convincing evidence of neurohormonal activation in virtually all patients with a Fontan circulation (including asymptomatic patients) (64). Similar observations have resulted in the development of- and evidence related to the use of ace inhibitors, angiotensin receptor blockers, mineralocorticoid receptor blockers, and betablockers in acquired heart failure, eventually improving outcome of those patients; however, studies to replicate these findings in the Fontan population have been negative until now.

Deterioration of the Fontan circulation also often coincides with an increased risk for thrombosis and thromboembolic events, which are frequent in Fontan patients, occurring in up to 33% of patients, and are often serious (mode of death in 25% of patients) (65–67). Several reports have indicated coagulation factor abnormalities, both prothrombotic (plasminogen deficiency, antithrombin 3 deficiency, and protein S and C defiency) as well as procoagulant (increase in factor VIII), which have been related to increased venous pressure (potentially affecting protein synthesis in the liver) (66). The contribution of inflammation, which has been observed in patients with a Fontan circulation, to thrombosis risk has not yet been investigated (68).

With limited patient numbers and insufficient power to perform adequate randomized controlled trials in Fontan patients, it may seem hopeless to even invest in similar research in Fontan patients. However, one could argue that a Fontan patient represents a prime example of a patient in a thrombo-inflammatory state with increased neurohormonal activation destined to develop diastolic dysfunction. A better understanding of the underlying pathomechanisms with identification of therapeutic targets may not only serve the Fontan patient, but the underserved HFpEF patient as well.

### Limitations

Further elaboration on the influence of arterio-venous and veno-venous collateral flow, the development of Fontan-associated liver disease, and abnormal lymphatics on Fontan hemodynamics is beyond the scope of this review, but should be considered in any patient with Fontan circulatory failure.

## Conclusions

The Fontan circulation provides a unique solution for patients with a single functional or anatomical ventricle, expanding life expectancy and quality of life. However, every Fontan circulation carries within itself the seeds of its own decay (69). Increased systemic venous pressures (and the subsequent development of Fontan-associated liver disease and abnormal lymphatics) and low cardiac output at rest, but especially during exercise (causing exercise intolerance), are the key components of every Fontan circulation and deteriorate slowly over time. The Fontan portal system redefines the place of the pulmonary circulation as the main factor influencing systemic venous return and hence cardiac output. This also infers that even small changes in atrial pressure could have large effects on cardiac output. Following this, the deleterious effects of diastolic dysfunction (accelerated aging, chronic deprivation), systolic dysfunction, atrioventricular valve regurgitation, and/or atrial dysfunction will be enhanced in the Fontan circulation. There is a responsibility for patients and caregivers alike during the life trajectory of a Fontan patient. The pediatric cardiologist and congenital cardiac surgeon should always aim for the perfect Fontan circulation, the patient should maintain an active, healthy lifestyle, avoiding weight gain, and the adult congenital cardiologist should not accept suboptimal hemodynamics (i.e., AV valve regurgitation, undersized Fontan conduits, or pulmonary artery hypoplasia). There is a need for innovation, such as retraining of the pulmonary vasculature and ventricle using right-sided assist devices prior to transplant, further research on implantable right-sided assist devices, aims to reduce the risk of AV valve surgery, and novel pathways to improve the thrombo-inflammatory state in Fontan patients.

## Author Contributions

AV: writing manuscript. GC and TS: critical review manuscript. MG: writing and critical review manuscript. All authors contributed to the article and approved the submitted version.

## Conflict of Interest

The authors declare that the research was conducted in the absence of any commercial or financial relationships that could be construed as a potential conflict of interest.

## Publisher's Note

All claims expressed in this article are solely those of the authors and do not necessarily represent those of their affiliated organizations, or those of the publisher, the editors and the reviewers. Any product that may be evaluated in this article, or claim that may be made by its manufacturer, is not guaranteed or endorsed by the publisher.
